# Reducing fatigue in pediatric rheumatic conditions: a systematic review

**DOI:** 10.1186/s12969-021-00580-8

**Published:** 2021-07-08

**Authors:** K. Kant-Smits, M. Van Brussel, S. Nijhof, J. Van der Net

**Affiliations:** 1 Department of Pediatrics, Wilhelmina Children’s Hospital, Center for Child Development Exercise and Physical Literacy, University Medical Center Utrecht, Utrecht University, Suite KB.02.055.1, Internal mail KB.02.056.0, PO Box 85090, 3508 AB Utrecht, The Netherlands; 2 Department of Pediatrics, Wilhelmina Children’s Hospital, University Medical Center Utrecht, Utrecht University, Utrecht, The Netherlands

**Keywords:** Fatigue, Systemic lupus erythematosus (cSLE), Juvenile dermatomyositis (JD), Juvenile idiopathic arthritis (JIA), Intervention, Children

## Abstract

**Background:**

Although fatigue is a prevalent distressing symptom in children and adolescents with Pediatric Rheumatic Conditions (PRCs), intervention studies designed for reducing fatigue in PRCs are limited.

**Aim:**

To systematically review evidence regarding the efficacy of interventions intended to reduce fatigue in patients with PRCs.

**Methods:**

Comprehensive electronic searches were performed in PubMed/ MEDLINE, Embase, Web of Science and Cinahl. The risk of bias was assessed using the ‘*Revised Cochrane risk-of-bias tool for randomized trials*’ and ‘*Quality Assessment Tool for Before-After Studies With No Control Group*’ for respectively studies with and without a control group.

**Results:**

Ten out of 418 studies were included with a total of 240 participants (age range 5–23 years). Interventions included land-based and aquatic-based exercise therapy, prednisolone, vitamin-D and creatine supplementation, psychological therapy and a transition program into an adult rheumatology program. Fatigue was assessed with self-reported questionnaires in all included studies. Land-based exercise therapy was effective in one pre-post intervention study, whereas not effective in two randomized controlled trials. Aquatic-based exercise therapy was found more effective than land-based exercise therapy. Two placebo-controlled studies showed a significant positive effect in reducing subjective fatigue with prednisolone and vitamin-D. Creatine was not found effective. Cognitive therapy was effective in one pre-post intervention study, while one RCT did not show an effect in reducing fatigue. A transition program based on health education showed a small reducing effect, however, it was not clear if this was a significant effect. Six studies showed a high risk of bias, three studies a moderate risk, and one study had a low risk of bias.

**Conclusions:**

Insufficient evidence is provided to substantiate the efficacy of current interventions to reduce fatigue in PRCs. The low number of studies, non-comparable interventions, risk of bias, and inconclusive outcomes of the included studies denote future research should focus on intervention studies aimed at the treatment of fatigue in children and adolescents with PRCs. Identification of possible underlying biological and psychosocial mechanisms as possible treatment targets to reduce complaints of fatigue in children and adolescents with PRCs is warranted.

**Supplementary Information:**

The online version contains supplementary material available at 10.1186/s12969-021-00580-8.

## Introduction

Pediatric Rheumatic Conditions (PRCs) are characterized by periods of disease flares and symptoms such as fatigue, stiffness, pain, muscle weakness, poor health-related quality of life, and/or difficulties performing routine activities at home and at school [1, 2]. The most common PRCs are Juvenile Idiopathic Arthritis (JIA) (incidence of 16–150/100,000) [3], Systemic Lupus Erythematosus with onset during childhood (cSLE) (incidence of 2.22/100,000) [4], and Juvenile Dermatomyositis (JDM) (incidence of 0.19–0.4/100,000) [5].

Fatigue is a profound and clinically frequently heard symptom in these pediatric conditions. The occurrence of fatigue has been reported in 60–76% of all children with JIA [6] and 30–74% in children with cSLE [7]. Furthermore, severe debilitating fatigue occurs in 25.1% of all adolescents with PRCs [1]. These occurrence numbers are significantly higher in children and adolescents with PRCs compared with healthy peers [1, 6]. Fatigue has been described as a multidimensional concept and is a subjective symptom in which perceived severity may be related to both psychosocial and disease- and treatment-related factors [8]. Several studies have suggested a relation of fatigue with pain, health-related quality of life (HRQoL), physical activity, medication, stress, mood, sleep dysregulation, increased school absences, and increased disease activity [1, 6, 8–10]. Most clinicians assume that fatigue is caused primarily by drugs such as Methotrexate or disease activity and inflammation in PRCs [9, 11]. However, fatigue can also persist in children independent of their disease-activity levels [1, 6, 10]. Apparently, fatigue can be better explained by transdiagnostic psychosocial factors – e.g. coping strategies; cognitive, emotional, and motivational factors; sleep and lifestyle)- rather than specific characteristics of the disease [12, 13]. Transdiagnostic can be defined as an approach in which clinicians aim to go beyond the disease-specific biological factors of disease and look for generic factors [14].

Many approaches have been studied regarding the management of musculoskeletal symptoms in PRCs, resulting in the development of PRC-specific guidelines [15–17]. However, these guidelines do not comprise the management of fatigue. Reviews regarding treatments and interventions primarily designed for reducing fatigue in children with PRCs are currently not available and were stated as an understudied area of interest by parents organizations and health providers at the 25th-anniversary meeting of the Paediatric Rheumatology European Association (PReS; 2019) [18]. Furthermore, the clinical significance of in depth information regarding fatigue is addressed twice in the top 10 research questions formulated for JIA according to the James Lind Alliance protocol [19, 20]. The comprehensive effects of fatigue demonstrate the relevance and importance of studying and designing interventions for children with a PRC. Notably, early detection and timely intervention may enhance the patients` well-being and their participation in daily life.

Therefore, our objective was to systematically review contemporary evidence regarding the efficacy of pharmacological and non-pharmacological interventions intended to reduce the severity of fatigue among pediatric patients with PRCs.

## Methods

For the conduct of this systematic review, we followed the Preferred Reporting Items for Systematic Reviews and Meta-analyses (PRISMA) statement [21].

### Search strategy

The electronic databases PubMed/Medline, Cochrane, Cinahl, Scopus, Embase and Pedro were searched for eligible articles up to December 2nd, 2020. The following MeSH terms and Emtree were employed: *“intervention”, “drug therapy”,*” *therapeutics”, “rehabilitation”, “exercise therapy”, “disease management”, “program”, “pediatric/paediatric rheumatic conditions”, “juvenile arthritis”, “systematic arthritis”, “juvenile rheumatoid arthritis”, “systemic lupus erythematosus”, “dermatomyositis”, “still’s disease”, “juvenile psoriatic arthritis”, “juvenile enthesitis-related arthritis”, “libman sacks disease”,, “fatigue”, “tiredness”, “lassitude”, “child”, “adolescent*” and “*clinical study”*. The complete search strategy can be found in Additional file 1: Appendix I.

### Inclusion and exclusion of studies

Eligibility criteria were defined a priori. Articles were eligible for inclusion if they were randomized or controlled intervention trials and 1) included children, adolescents, and young adults with JIA, cSLE or JDM (most three common types of PRC); and 2) assessed fatigue as a primary or secondary outcome. The search did not have language restrictions. Articles were excluded if they 1) were study protocols or abstracts and 2) were not available in full text.

### Study selection

**Fig. 1:**
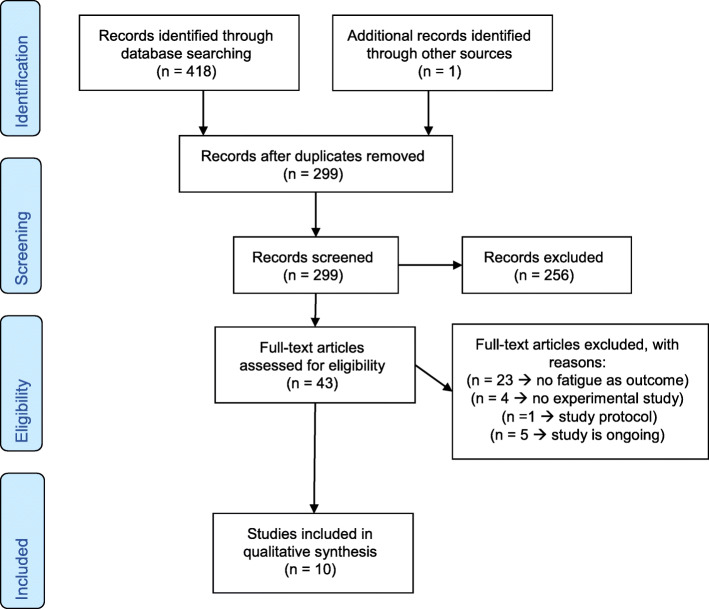
Flow diagram of the article selection search

Initially, articles were screened for eligibility based on their title and abstract. When the title and abstract implied that an article was potentially eligible for inclusion, a full paper copy of the report was obtained. If there was any doubt regarding the articles` eligibility, the article inclusion was discussed with the review group until consensus was reached. Additionally, reference tracking and snowballing were performed in all included articles (see Fig. 1: flowchart).

### Data extraction and management

The characteristics of the included studies were extracted using a standard extraction form. Data extracted from the included studies were: 1) first author, publication year, and the location of the study; 2) used intervention and type of PRC; 3) study design; 4) number and age of participants; 5) type of intervention and control; 6) measurement used for fatigue; 6) study results and 7) conclusion regarding fatigue. If data were missing or further information was required, serious attempts were made to contact the corresponding authors. Additionally, the conclusions of the methodological quality assessment were added to provide an instant overview. The results of the data extraction are depicted in Table 1.

**Table 1:**
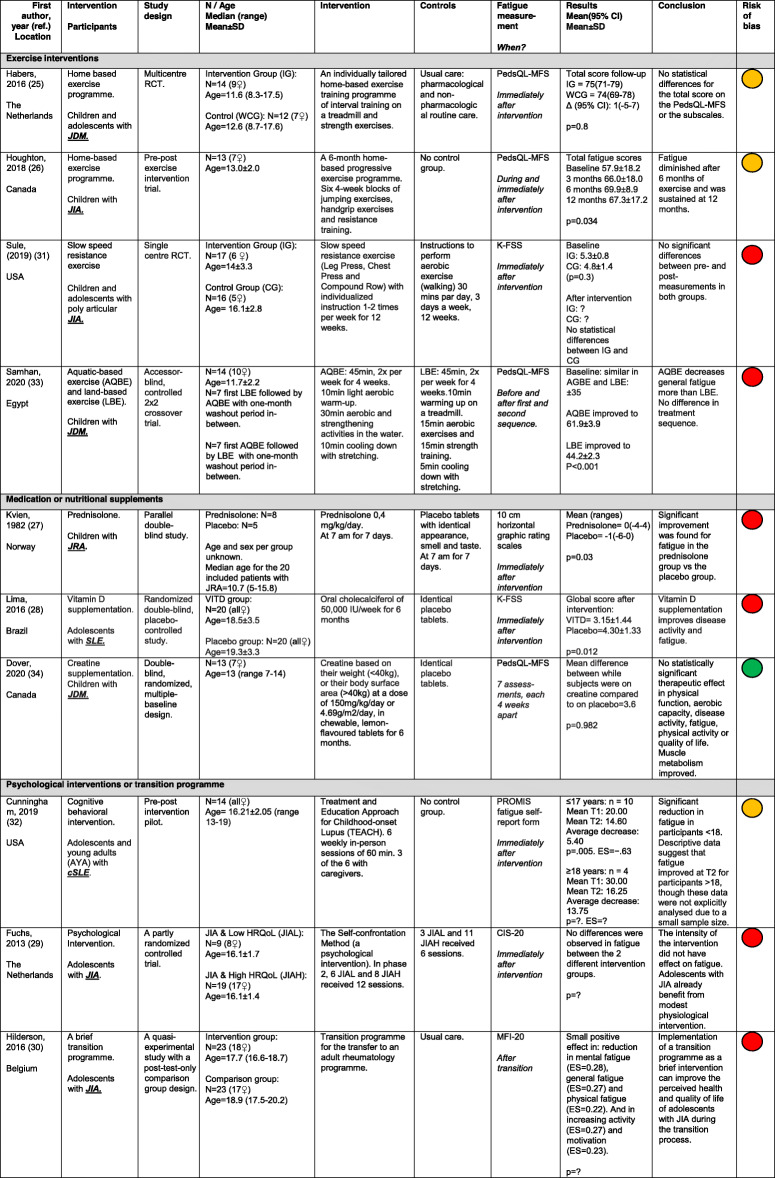
Descriptives and characteristics of the included articles (*n* = 10

? Unknown/not shown, ♀ Girl/woman, *CI* Confidence interval, *CIS-20* Checklist for Individual Strength, *ES* Effect size, *HRQoL* Health related quality of life, *JDM* Juvenile dermatomyositis, *JIA* Juvenile idiopathic arthritis, *JRA* Juvenile rheumatoid arthritis, *kg* Kilogram, *K-FSS* Kids Fatigue Severity Scale, *MFI-20* Multidimensional Fatigue Inventory, *min* Minutes, *N* Number, *p* estimated probability, significant if *p* < 0,05, *PedsQL- MFS* Pediatric Quality of Life Inventory - Multidimensional Fatigue Scale, *RCT* Randomized controlled trial, *SD* Standard deviation, *SLE* Systematic lupus erythematosus, *VITD* Vitamin D supplementation group. : high risk of bias, : some concerns/moderate risk of bias, : low risk of bias

### Assessment of methodological quality

Two reviewers (KK & JvdN) independently assessed the methodological quality of the included articles. The Cochrane Collaboration’s instrument for assessing the risk of bias in randomized trials was used [22] See Additional file 1: Appendix II. Elements evaluated were adequate sequence generation, adequate allocation concealment, blinding of participants and personnel, blinding of outcome assessors, and lack of attrition bias [22]. To compare the methodological quality of the articles, this tool has also been used for studies without randomization, but with a control group. The methodological quality of studies without a control group was scored using the ‘Quality Assessment Tool for Before-After (Pre-Post) Studies With No Control Group’ [23]. See Additional file 1: Appendix III. The questions in this quality assessment tool were designed to help reviewers focus on the key concepts for evaluating the internal validity of a study. The tool consists of 12 questions, which can be answered with yes, no, cannot determine, not applicable, or not reported. For the conclusion, the ‘yes-scores’ of the criteria can be added together and divided by 12 [23]. Scores between 75 and 100% were considered as low risk of bias, scores between 25 and 75% were considered as moderate risk of bias, and scores between 0 and 25% were considered as high risk of bias. The strength of inter-rater agreement after the first scoring was measured by Cohen’s κ coefficient with κ = 0.41–0.60 indicating moderate agreement, κ = 0.61–0.80 representing good agreement, and κ ≥ 0.81 representing very good agreement [24, 25]. Disagreements were resolved in a consensus meeting between the raters.

### Data synthesis

Results were reported descriptively. Meta-analysis was not applicable due to the heterogeneity of the studies. To synthesize the quality scores, the rating system of Proper et al. [26] was applied. This rating system takes into account the number, the quality, and the outcome of the studies. The conclusions were drawn based on the following three levels of scientific evidence:

1. Strong evidence: consistent findings in multiple (> 2) high-quality studies;
2. Moderate evidence: consistent findings in one high-quality study and one or more low-quality studies, or multiple low-quality studies;
3. Insufficient evidence: only one study available or inconsistent findings in multiple (> 2) studies.

Similar to previous reviews that applied this best evidence synthesis, results were considered to be consistent when at least 75% of the studies showed results in the same direction, which was defined according to significance (*p* < 0.05). If there were two or more high quality studies, the studies of low methodologic quality were disregarded in the evidence synthesis [26].

## Results

### Study selection

Figure 1 shows the flow diagram of study identification and selection and reasons for excluding studies. The search strategy identified 418 records of which in total, ten studies [27–36] with a total of 240 patients were included for review.

### Study characteristics

Table 1 shows the demographics of the included studies. Five studies were Randomized Controlled Trials (RCT) [27, 30, 31, 33, 36], two studies were controlled trials [29, 35], two studies were a pre-post intervention trial [28, 34] and one study was a quasi-experimental study with a post-test-only comparison group design [32]. Four of the ten studies included children or adolescents with JIA (total *n* = 120) [28, 31–33], two studies included adolescents with cSLE (*n* = 34) [30, 34], three studies included children and adolescents with JDM (*n* = 26) [27, 35, 36] and one study used the more general (and older) term juvenile rheumatic arthritis (JRA) (*n* = 13) [29]. Three studies evaluated land-based exercise therapy as their intervention [27, 28, 33], one study used aquatic-based exercise therapy [36], three studies evaluated medication/nutritional supplements as an intervention [29, 30, 35], two studies examined a psychological/cognitive behavioral intervention [31, 34] and one study investigated the results of a transition program into an adult rheumatology program [32]. In all included studies fatigue was assessed with a self-report questionnaire. Four studies used the Pediatric Quality of Life Multidimensional fatigue scale (PEDsQL-MFS) [27, 28, 35, 36], two studies used the Kids Fatigue Severity Scale (K-FSS) [30, 33], one the Multidimensional Fatigue Inventory (MFI-20) [32], one the Checklist for Individual Strength (CIS-20) [31], one the PROMIS fatigue self-report form [34] and one a ten cm visual analogue scale (VAS) [29]. In all studies the sample size was small (five to 23 participants per group). In only one study the required sample size was calculated and achieved [27]. In line with the expected characteristics of the population with PRCs, studies included predominantly female participants (mean 73%, range: 34–100%) with mean age ranging from 10.7 to 19.3 years across all studies. Further study characteristics of the included studies, as reported in these articles, are described in Table 1.

### Methodological quality

The methodological quality of eight [27, 29–33, 35, 36] of the included articles was assessed by using the RoB 2 [22]. See Table 1 for the conclusion of the methodological quality assessment and Additional file 1: Appendix IV for a more detailed risk of bias summary. The Cohen’s κ coefficient was 0.69 (*p* = 0.000), 95% CI (0.50, 0.88) for the raters, representing good agreement. Six studies received the judgment of high risk of bias [29–33, 35]. One study [27] was judged to have a moderate risk of bias and only one study had a low risk of bias [36].

The methodological quality of two studies [28, 34] was assessed by using the ‘Quality Assessment Tool for Before-After (Pre-Post) Studies With No Control Group’ [23]. See Table 1 for the conclusion of the methodological quality assessment and Additional file 1: Appendix V for a more detailed risk of bias summary. The Cohen’s κ coefficient was 0.70 (*p* = 0.000), 95% CI (0.47, 0.94) for the two raters, representing good agreement. There was a moderate risk of bias in both studies.

After the first scoring, there was already a good agreement between the two raters. There were minor disagreements, mainly caused by interpretation differences, which were resolved in a consensus meeting by discussing them without causing a methodological bias.

### Synthesis of results

After summarizing the included studies, it appeared that the studies were very heterogeneous, especially concerning the type of intervention and assessment of fatigue. The results of the individual studies can be found in Additional file 1: Appendix VI. The different types of interventions were combined in three categories: exercise therapy, medication/nutritional supplements, and psychological intervention/transition program. The three categories of interventions were analyzed with the best-evidence synthesis of Proper et al. [26].

There is insufficient evidence for the effectiveness of (home-based) land-based exercise therapy to reduce fatigue in children and adolescents with PRCs as there were inconsistent findings in multiple studies. Although Houghton et al. [28] found a significant improvement in fatigue during and after a 6-month exercise program (fatigue scores improved by 8.1 points, 12.0 points and 9.4 points, on average, at 3, 6 and 12 months, respectively (*p* = 0.034)), the studies of Habers et al. [27] and Sule et al. [33] did not found a significant improvement in fatigue immediately after a 12 weeks exercise training program. Aquatic-based exercise therapy was evaluated in only one study [35] and improved fatigue more than land-based exercises (improvement to 61.9 ± 3.9 and 44.2 ± 2.3, *p* < 0.001).

Two of the interventions in the category of medication/nutritional supplements were effective in reducing fatigue [29, 30]. Significant improvement in fatigue was found after receiving 6 months of vitamin-D supplementation (*p* = 0.012) [30] or 7 days of prednisolone (*p* = 0.03) [29]. However, both studies had a high risk of bias and the two interventions were not comparable. Creatine supplementation was not effective in reducing fatigue (*p* = 0.982) [36]. Therefore, synthesis indicates insufficient evidence for the effectiveness of the use of prednisolone, vitamin-D supplementation, and/or creatine supplementation to reduce fatigue in children and adolescents with PRCs.

Fuchs et al. [31] and Cunningham et al. [34] both used a psychological intervention to reduce fatigue in adolescents with PRCs (resp. ‘The Self-confrontation Method’ and “Treatment and Education Approach’). Fuchs et al. found no significant difference at the end of the intervention between the group who received six sessions and the group who received 12 sessions of the psychological intervention. They stated that the intensity of the intervention did not influence the outcome measures. In general, adolescents with JIA already benefit from modest psychological intervention [31]. Cunningham et al. found a statistically significant reduction in fatigue (average decrease of 5.40, *p* = 0.005, effect size (ES) = − 0.63) directly after completing the intervention [34]. Hilderson et al. found a small positive effect (ES = 0.27, p = not shown) in general fatigue measured after the transfer, following a transition program based on family-centered health education into an adult rheumatology program [32]. The interventions used in the abovementioned studies were not comparable and the studies were of low quality. Therefore, there is also insufficient evidence for the effectiveness of psychological therapy with the Self-confrontation method or cognitive behavioral therapy with the Treatment and Education Approach for Childhood-onset Lupus (TEACH) protocol or a transition program based on health education to the adult rheumatology program in reducing fatigue in children and adolescents with PRCs.

## Discussion

Fatigue is a prevalent distressing symptom in children and adolescents with PRCs [1, 13] and can have a significant impact on the well-being and participation in the daily life of the patient and his or her family [37–39]. The ability to early and adequately assess, treat and reduce the severity of fatigue can improve their current well-being and participation in daily life, as well as their future well-being by preventing fatigue from becoming persistent [13, 40, 41]. Despite the high impact and long-lasting consequences of fatigue in patients with PRC, current therapeutic options are limited. The strikingly few studies regarding the efficacy of interventions intended to reduce fatigue in patients with PRCs suggested a small but significant effect in the individual studies, although the evidence syntheses indicate insufficient evidence to substantiate the efficacy of current interventions to reduce the severity of fatigue in PRCs. In most of the studies, fatigue was not the primary outcome and none of the studies controlled for disease activity or stated that the study population was well-controlled with overall low disease activity. The low number of studies, heterogeneity in diagnostic groups, ages and disease stages, heterogeneity in the type of interventions, risk of bias, and inconclusive outcomes of the included studies entangles to provide a general statement regarding the efficacy of the applied interventions and especially regarding possible superiority of one type of therapy. Our current results underline the need for more multidimensional intervention studies primarily aimed at the treatment of fatigue in children and adolescents with PRCs and the need for increased awareness to measure fatigue accurately and regularly in this patient group during treatment and follow-up.

Physical and psychological therapy are considered the most promising treatment approaches in reducing fatigue in adults with Rheumatoid Arthritis (RA) [42]. Cramp et al. [42] concluded, based on a meta-analysis of six studies (388 participants) investigating physical therapy (such as pool-based therapy, yoga, strength training, and aerobics) and 13 studies (1579 participants) investigating psychological interventions (such as cognitive-behavioral therapy (CBT), mindfulness, self-management and group education), that physical activity and psychological interventions showed a small statistically significant effect in reducing fatigue in adults with RA. Hewlet et al. [43, 44] recently described a positive effect of group CBT on reducing fatigue impact in patients with RA, compared with receiving fatigue information alone. CBT might also be applicable for patients with PRCs although further research is warranted.

All before mentioned reviews state, similar to this review, that the included studies had heterogeneous interventions, with small sample sizes, different outcome measures, and intervention protocols and therefore do not lead to conclusive evidence.

The strength of this review is its focus on interventions to target fatigue in patients with PRCs. However, meta-analysis was not possible with the data reported by the studies found that met the inclusion criteria. The limited number of included studies are small, diverse, and inconclusive. Furthermore, there was wide variation in intervention procedures, study designs and outcome-measuring instruments among the individual studies and because of the limited number of studies available for synthesis, stratified analysis was not possible. Therefore, it is not possible to give recommendations for the implementation of effective interventions in daily practice in children with PRCs.

Fatigue is probably not only a biological (side-)effect of PRCs and its treatment but also the complex result of the physical and psychosocial challenges of growing up with a chronic disease [13, 14, 45]. Therefore, the answer might have to be sought in a multidimensional cause and a dynamic interplay between mind and body. The demonstrated interventions all focused on different (single) possible causes of fatigue in children and adolescents with different types of PRCs. The conceptual model on fatigue in JIA presented by Armbrust et al. [6], see Additional file 1: Appendix VII, illustrates that fatigue seems multifactorial and should therefore be approached and treated as such. The model illustrates a theoretical interplay of ‘disease-related’, ‘personal’ factors and ‘environmental’, ‘generic’ factors that could contribute to the origin and maintenance of fatigue in PRCs. We, therefore, suggest that interventions should focus on both the physical and psychosocial aspects of fatigue, as, for example, in CBT [46]. CBT has been proven to reduce fatigue in adolescents with chronic fatigue syndrome (CFS), and in fatigued adults with various chronic diseases including RA [43, 44, 46–48]. Other promising interventions include education, exercise, healing touch, or relaxation [37, 49–52]. Physical activity may be effective in reducing fatigue by decreasing inflammation, increasing muscle strength or mass, and improving functional capacity and mental health [53, 54].

In conclusion, there is an urgent need for more intervention studies that primary aim at the treatment of fatigue in children and adolescents with PRCs. Identification of possible underlying biological and psychosocial mechanisms as possible treatment targets to reduce complaints of fatigue in children and adolescents with PRCs is warranted. Future studies should investigate interventions applying a multifactorial approach of fatigue, and therefore be aimed at the physical and psychosocial dimensions to fatigue, combined with an assessment to determine fatigue in relation to physical, environmental, and personal outcome parameters [6, 10, 15].

## Supplementary Information

**Additional file 1: Appendix I**. Search strings. **Appendix II.** Revised Cochrane risk-of-bias tool for randomized trials (RoB 2.0). **Appendix III.** *The quality assessment tool for before-after (pre-post) studies with no control group.* **Appendix IV.** Risk of bias summary: review authors’ judgements about each risk of bias item for each included controlled study. **Appendix V.** Risk of bias summary: the quality assessment scores of the included pre-post studies. **Appendix VI.** Results of individual studies. **Appendix VII.** Conceptual model of fatigue in patients with JIA [6].

## Data Availability

NA

## Abbreviations

♂: Girl/woman
CD: Cannot determine
CI: Confidence interval
CIS-20: Checklist for Individual Strength
cSLE: Systematic Lupus Erythematosus with onset in childhood
CT: Controlled trial
ES: Effect size
HRQoL: Health related quality of life
JDM: Juvenile dermatomyositis
JIA: Juvenile idiopathic arthritis
JRA: Juvenile rheumatoid arthritis
κ: Kappa
K-FSS: Kids Fatigue Severity Scale
MeSH: Medical Subject Headings
MFI-20: Multidimensional Fatigue Inventory
N/n: Number
N: No
NA: Not applicable
NR: Not reported
p: Estimated probability, significant if *p* < 0,05
PedsQL- MFS: Pediatric Quality of Life Inventory - Multidimensional Fatigue Scale
PRCs: Paediatric Rheumatic Conditions
PReS: Paediatric Rheumatology European Association
PRISMA: Preferred reporting for systematic reviews and meta-analysis
RA: Rheumatoid arthritis
RCT: Randomized controlled trial
ref.: Reference
RoB 2: Revised Cochrane risk-of-bias tool for randomized trials
SD: Standard deviation
SLE: Systematic lupus erythematosus
VITD: Vitamin D supplementation group
Y: Yes
High risk of bias
Some concerns/moderate risk of bias
Low risk of bias
