# Supplementary material for: Reducing fatigue in pediatric rheumatic conditions: a systematic review
Source: Pediatr Rheumatol Online J. 2021 Jul 8;19:111. doi: 10.1186/s12969-021-00580-8 (PMC8268602; doi:10.1186/s12969-021-00580-8)
Supplement: Supplementary file 1 — Additional file 1: Appendix I. Search strings. Appendix II. Revised Cochrane risk-of-bias tool for randomized trials (RoB 2.0). Appendix III. The quality assessment tool for before-after (pre-post) studies with no control group. Appendix IV. Risk of bias summary: review authors’ judgements about each risk of bias item for each included controlled study. Appendix V. Risk of bias summary: the quality assessment scores of the included pre-post studies. Appendix VI. Results of individual studies. Appendix VII. Conceptual model of fatigue in patients with JIA [6]. [file 12969_2021_580_MOESM1_ESM.docx]

Appendix I: Search strings

**PubMed/Medline**

((("drug therapy"[Mesh] OR "therapeutics"[Mesh] OR "rehabilitation"[Mesh] OR "exercise"[Mesh] OR "disease management"[Mesh] OR "exercise therapy"[Mesh]) OR ("strategies"[Title/Abstract] OR "response"[Title/Abstract] OR "remedy"[Title/Abstract] OR "regime"[Title/Abstract] OR "recovery"[Title/Abstract] OR "program"[Title/Abstract] OR "programs"[Title/Abstract] OR "management"[Title/Abstract] OR "manage"[Title/Abstract] OR "intervention"[Title/Abstract] OR "help"[Title/Abstract] OR "healing"[Title/Abstract] OR "doctoring"[Title/Abstract] OR "curing"[Title/Abstract] OR "cure"[Title/Abstract] OR "care"[Title/Abstract] OR "aid"[Title/Abstract] OR "training"[Title/Abstract] OR "trainings"[Title/Abstract] OR "Treatments"[Title/Abstract] OR "Treatment"[Title/Abstract] OR "Therapies"[Title/Abstract] OR "Therapy"[Title/Abstract] OR "therapeutic"[Title/Abstract] OR "therapeutics"[Title/Abstract] OR "habilitation"[Title/Abstract] OR "rehabilitation"[Title/Abstract] OR "Procedure"[Title/Abstract] OR "procedures"[Title/Abstract] OR "Exercise Rehabilitation"[Title/Abstract] OR "Exercises Rehabilitation"[Title/Abstract] OR "Rehabilitation Exercise"[Title/Abstract] OR "Rehabilitation Exercises"[Title/Abstract] OR "Therapies Exercise"[Title/Abstract] OR "Exercise Therapies"[Title/Abstract] OR "Therapy Exercise"[Title/Abstract] OR "Remedial Exercise"[Title/Abstract] OR "Remedial Exercises"[Title/Abstract] OR "exercise therapy"[Title/Abstract] OR "Training Exercise"[Title/Abstract] OR "Exercise Training"[Title/Abstract] OR "Exercise Trainings"[Title/Abstract] OR "Aerobic Exercise"[Title/Abstract] OR "Aerobic Exercises"[Title/Abstract] OR "Exercise Aerobic"[Title/Abstract] OR "Exercises Aerobic"[Title/Abstract] OR "Isometric Exercise"[Title/Abstract] OR "Isometric Exercises"[Title/Abstract] OR "Exercise Isometric"[Title/Abstract] OR "Exercises Isometric"[Title/Abstract] OR "Exercise Acute"[Title/Abstract] OR "Acute Exercise"[Title/Abstract] OR "Acute Exercises"[Title/Abstract] OR "Physical Exercise"[Title/Abstract] OR "Physical Exercises"[Title/Abstract] OR "Exercise Physical"[Title/Abstract] OR "Exercises Physical"[Title/Abstract] OR "Physical Activities"[Title/Abstract] OR "Activity Physical"[Title/Abstract] OR "Physical Activity"[Title/Abstract] OR "Exercises"[Title/Abstract] OR "exercise"[Title/Abstract] OR "Management Disease"[Title/Abstract] OR "Disease managements"[Title/Abstract] OR "disease management"[Title/Abstract] OR "drug therapy"[Title/Abstract] OR "therapy drug"[Title/Abstract] OR "drug therapies"[Title/Abstract] OR "Pharmacotherapy"[Title/Abstract] OR "Pharmacotherapies"[Title/Abstract] OR "intervention"[Title/Abstract] OR "trial"[Title/Abstract] **OR "mindfulness"[Title/Abstract] OR "cognitive behavioral therapy"[Title/Abstract] OR "cognitive-behavioral therapy"[Title/Abstract] OR "self-management"[Title/Abstract] OR "psychotherapy"[Title/Abstract] OR "psychological treatment"[Title/Abstract] OR "medication"[Title/Abstract]**)) AND (("Arthritis, Juvenile"[Mesh] OR "Lupus Erythematosus, Systemic"[Mesh] OR "Dermatomyositis"[Mesh]) OR ("arthritis juvenile"[Title/Abstract] OR "juvenile arthritis"[Title/Abstract] OR "Arthritis Juvenile Chronic"[Title/Abstract] OR "Chronic Arthritis Juvenile"[Title/Abstract] OR "Juvenile Rheumatoid Arthritis"[Title/Abstract] OR "Juvenile Chronic Arthritis"[Title/Abstract] OR "Arthritis Juvenile Rheumatoid"[Title/Abstract] OR "Rheumatoid Arthritis Juvenile"[Title/Abstract] OR "Juvenile Idiopathic Arthritis"[Title/Abstract] OR "Still's Disease"[Title/Abstract] OR "Still Disease"[Title/Abstract] OR "Systemic Arthritis"[Title/Abstract] OR "Arthritis Systemic"[Title/Abstract] OR "Juvenile Systemic Arthritis"[Title/Abstract] OR "Stills Disease"[Title/Abstract] OR "Juvenile Psoriatic Arthritis"[Title/Abstract] OR "Juvenile Enthesitis-Related Arthritis"[Title/Abstract] OR "Libman Sacks Disease"[Title/Abstract] OR "Libman-Sacks Disease"[Title/Abstract] OR "Lupus Erythematosus Disseminatus"[Title/Abstract] OR "Systemic Lupus Erythematosus"[Title/Abstract] OR "Lupus Erythematosus Systemic"[Title/Abstract] OR "Juvenile Myositis"[Title/Abstract] OR "Dermatopolymyositis"[Title/Abstract] OR "Dermatomyositis Juvenile"[Title/Abstract] OR "Juvenile Dermatomyositis"[Title/Abstract] OR "Childhood Type Dermatomyositis"[Title/Abstract] OR "Polymyositis Dermatomyositis"[Title/Abstract] OR "Dermatomyositis"[Title/Abstract] OR "Polymyositis-Dermatomyositis"[Title/Abstract] OR "Pediatric Rheumatic Conditions"[Title/Abstract]))) AND ("Tiredness"[Title/Abstract] OR "lassitude"[Title/Abstract] OR "fatigue"[Title/Abstract]) AND (Clinical Study[ptyp] AND ("infant"[MeSH Terms] OR "child"[MeSH Terms] OR "adolescent"[MeSH Terms]))

**Cochrane**

**#1 intervention**

[mh “Drug therapy”] OR [mh "Therapeutics"] OR [mh "Rehabilitation"] OR [mh "Exercise"] OR [mh "Disease Management"] OR [mh "Exercise Therapy"] OR “strategies”:ti,ab,kw OR “response”:ti,ab,kw OR “remedy”:ti,ab,kw OR “regime”:ti,ab,kw OR “recovery”:ti,ab,kw OR “program”:ti,ab,kw OR “programs”:ti,ab,kw OR “management”:ti,ab,kw OR “manage”:ti,ab,kw OR “intervention”:ti,ab,kw OR “help”:ti,ab,kw OR “healing”:ti,ab,kw OR “doctoring”:ti,ab,kw OR “curing”:ti,ab,kw OR “cure”:ti,ab,kw OR “care”:ti,ab,kw OR “aid”:ti,ab,kw OR “training”:ti,ab,kw OR “trainings”:ti,ab,kw OR “Treatments”:ti,ab,kw OR “Treatment”:ti,ab,kw OR “Therapies”:ti,ab,kw OR “Therapy”:ti,ab,kw OR “therapeutic”:ti,ab,kw OR “therapeutics”:ti,ab,kw OR “habilitation”:ti,ab,kw OR “rehabilitation”:ti,ab,kw OR “Procedure”:ti,ab,kw OR “procedures”:ti,ab,kw OR “Exercise Rehabilitation”:ti,ab,kw OR “Exercises Rehabilitation”:ti,ab,kw OR “Rehabilitation Exercise”:ti,ab,kw OR “Rehabilitation Exercises”:ti,ab,kw OR “Therapies Exercise”:ti,ab,kw OR “Exercise Therapies”:ti,ab,kw OR “Therapy Exercise”:ti,ab,kw OR “Remedial Exercise”:ti,ab,kw OR “Remedial Exercises”:ti,ab,kw OR “exercise therapy”:ti,ab,kw OR “Training Exercise”:ti,ab,kw OR “Exercise Training”:ti,ab,kw OR “Exercise Trainings”:ti,ab,kw OR “Aerobic Exercise”:ti,ab,kw OR “Aerobic Exercises”:ti,ab,kw OR “Exercise Aerobic”:ti,ab,kw OR “Exercises Aerobic”:ti,ab,kw OR “Isometric Exercise”:ti,ab,kw OR “Isometric Exercises”:ti,ab,kw OR “Exercise Isometric”:ti,ab,kw OR “Exercises Isometric”:ti,ab,kw OR “Exercise Acute”:ti,ab,kw OR “Acute Exercise”:ti,ab,kw OR “Acute Exercises”:ti,ab,kw OR “Physical Exercise”:ti,ab,kw OR “Physical Exercises”:ti,ab,kw OR “Exercise Physical”:ti,ab,kw OR “Exercises Physical”:ti,ab,kw OR “Physical Activities”:ti,ab,kw OR “Activity Physical”:ti,ab,kw OR “Physical Activity”:ti,ab,kw OR “Exercises”:ti,ab,kw OR “exercise”:ti,ab,kw OR “Management Disease”:ti,ab,kw OR “Disease Managements”:ti,ab,kw OR “disease management”:ti,ab,kw OR “drug therapy”:ti,ab,kw OR “therapy drug”:ti,ab,kw OR “drug therapies”:ti,ab,kw OR “Pharmacotherapy”:ti,ab,kw OR “Pharmacotherapies”:ti,ab,kw OR "intervention":ti,ab,kw OR "trial":ti,ab,kw OR **"mindfulness"**:ti,ab,kw OR **"cognitive behavioral therapy"**:ti,ab,kw **OR "cognitive-behavioral therapy"**:ti,ab,kw **OR "self-management"**:ti,ab,kw **OR "psychotherapy"**:ti,ab,kw **OR "psychological treatment"**:ti,ab,kw **OR "medication"**:ti,ab,kw

**#2 Patients**

[mh "Lupus Erythematosus, Systemic"] OR [mh "Dermatomyositis"] OR [mh "Arthritis, Juvenile"] OR “Libman Sacks Disease”:ti,ab,kw OR “Libman-Sacks Disease”:ti,ab,kw OR “Lupus Erythematosus Disseminatus”:ti,ab,kw OR “Systemic Lupus Erythematosus”:ti,ab,kw OR “Lupus Erythematosus Systemic”:ti,ab,kw OR “Juvenile Myositis”:ti,ab,kw OR “Dermatopolymyositis”:ti,ab,kw OR “Dermatomyositis Juvenile”:ti,ab,kw OR “Juvenile Dermatomyositis”:ti,ab,kw OR “Childhood Type Dermatomyositis”:ti,ab,kw OR “Polymyositis Dermatomyositis”:ti,ab,kw OR “Dermatomyositis”:ti,ab,kw OR “Polymyositis-Dermatomyositis”:ti,ab,kw OR “Pediatric Rheumatic Conditions”:ti,ab,kw OR “Juvenile Psoriatic Arthritis”:ti,ab,kw OR “Juvenile Enthesitis-Related Arthritis”:ti,ab,kw OR “arthritis juvenile”:ti,ab,kw OR “juvenile arthritis”:ti,ab,kw OR “Arthritis Juvenile Chronic”:ti,ab,kw OR “Chronic Arthritis Juvenile”:ti,ab,kw OR “Juvenile Rheumatoid Arthritis”:ti,ab,kw OR “Juvenile Chronic Arthritis”:ti,ab,kw OR “Arthritis Juvenile Rheumatoid”:ti,ab,kw OR “Rheumatoid Arthritis Juvenile”:ti,ab,kw OR “Juvenile Idiopathic Arthritis”:ti,ab,kw OR “Still Disease”:ti,ab,kw OR “Systemic Arthritis”:ti,ab,kw OR “Arthritis Systemic”:ti,ab,kw OR “Juvenile Systemic Arthritis”:ti,ab,kw OR “Stills Disease”:ti,ab,kw

**#3 Outcome**

“Tiredness”:ti,ab,kw OR “lassitude”:ti,ab,kw OR “fatigue”:ti,ab,kw

**#4 Patients 2**

[mh child] OR [mh adolescent] OR [mh "preschool child"] OR “child”:ti,ab,kw OR “adolescent”:ti,ab,kw OR “children”:ti,ab,kw OR “child, preschool”:ti,ab,kw OR “juvenile”:ti,ab,kw OR “paediatric”:ti,ab,kw OR “pediatric”:ti,ab,kw OR “childhood”:ti,ab,kw

#1 AND #2 AND #3 AND #4.

**Cinahl**

**#1 Intervention**

TI OR AB OR SU((strategies) OR (response) OR (remedy) OR (regime) OR (recovery) OR (program) OR (programs) OR (management) OR (manage) OR (intervention) OR (help) OR (healing) OR (doctoring) OR (curing) OR (cure) OR (care) OR (aid) OR (training) OR (trainings) OR (Treatments) OR (Treatment) OR (Therapies) OR (Therapy) OR (therapeutic) OR (therapeutics) OR (habilitation) OR (rehabilitation) OR (Procedure) OR (procedures) OR (Exercise Rehabilitation) OR (Exercises Rehabilitation) OR (Rehabilitation Exercise) OR (Rehabilitation Exercises) OR (Therapies Exercise) OR (Exercise Therapies) OR (Therapy Exercise) OR (Remedial Exercise) OR (Remedial Exercises) OR (exercise therapy) OR (Training Exercise) OR (Exercise Training) OR (Exercise Trainings) OR (Aerobic Exercise) OR (Aerobic Exercises) OR (Exercise Aerobic) OR (Exercises Aerobic) OR (Isometric Exercise) OR (Isometric Exercises) OR (Exercise Isometric) OR (Exercises Isometric) OR (Exercise Acute) OR (Acute Exercise) OR (Acute Exercises) OR (Physical Exercise) OR (Physical Exercises) OR (Exercise Physical) OR (Exercises Physical) OR (Physical Activities) OR (Activity Physical) OR (Physical Activity) OR (Exercises) OR (exercise) OR (Management Disease) OR (Disease Managements) OR (disease management) OR (drug therapy) OR (therapy drug) OR (drug therapies) OR (Pharmacotherapy) OR (Pharmacotherapies) OR (intervention) OR (mindfulness) OR (cognitive behavioral therapy) OR (cognitive-behavioral therapy) OR (self-management) OR (psychotherapy) OR (psychological treatment) OR (medication))

**#2 Patients**

TI OR AB OR SU((Libman Sacks Disease) OR (Libman-Sacks Disease) OR (Lupus Erythematosus Disseminatus) OR (Systemic Lupus Erythematosus) OR (Lupus Erythematosus Systemic) OR (Juvenile Myositis) OR (Dermatopolymyositis) OR (Dermatomyositis Juvenile) OR (Juvenile Dermatomyositis) OR (Childhood Type Dermatomyositis) OR (Polymyositis Dermatomyositis) OR (Dermatomyositis) OR (Polymyositis-Dermatomyositis) OR (Pediatric Rheumatic Conditions) OR (Juvenile Psoriatic Arthritis) OR (Juvenile Enthesitis-Related Arthritis) OR (arthritis juvenile) OR (juvenile arthritis) OR (Arthritis Juvenile Chronic) OR (Chronic Arthritis Juvenile) OR (Juvenile Rheumatoid Arthritis) OR (Juvenile Chronic Arthritis) OR (Arthritis Juvenile Rheumatoid) OR (Rheumatoid Arthritis Juvenile) OR (Juvenile Idiopathic Arthritis) OR (Still Disease) OR (Systemic Arthritis) OR (Arthritis Systemic) OR (Juvenile Systemic Arthritis) OR (Stills Disease))

**#3 Outcome**

TI OR AB ((Tiredness) OR (lassitude) OR (fatigue))

#1 AND #2 AND #3 AND All child AND journal article

**Scopus**

**#1 Intervention**

TITLE-ABS-KEY(strategies) OR TITLE-ABS-KEY(response) OR TITLE-ABS-KEY(remedy) OR TITLE-ABS-KEY(regime) OR TITLE-ABS-KEY(recovery) OR TITLE-ABS-KEY(program) OR TITLE-ABS-KEY(programs) OR TITLE-ABS-KEY(management) OR TITLE-ABS-KEY(manage) OR TITLE-ABS-KEY(intervention) OR TITLE-ABS-KEY(help) OR TITLE-ABS-KEY(healing) OR TITLE-ABS-KEY(doctoring) OR TITLE-ABS-KEY(curing) OR TITLE-ABS-KEY(cure) OR TITLE-ABS-KEY(care) OR TITLE-ABS-KEY(aid) OR TITLE-ABS-KEY(training) OR TITLE-ABS-KEY(trainings) OR TITLE-ABS-KEY(Treatments) OR TITLE-ABS-KEY(Treatment) OR TITLE-ABS-KEY(Therapies) OR TITLE-ABS-KEY(Therapy) OR TITLE-ABS-KEY(therapeutic) OR TITLE-ABS-KEY(therapeutics) OR TITLE-ABS-KEY(habilitation) OR TITLE-ABS-KEY(rehabilitation) OR TITLE-ABS-KEY(Procedure) OR TITLE-ABS-KEY(procedures) OR TITLE-ABS-KEY(“Exercise Rehabilitation”) OR TITLE-ABS-KEY(“Exercises Rehabilitation”) OR TITLE-ABS-KEY(“Rehabilitation Exercise”) OR TITLE-ABS-KEY(“Rehabilitation Exercises”) OR TITLE-ABS-KEY(“Therapies Exercise”) OR TITLE-ABS-KEY(“Exercise Therapies”) OR TITLE-ABS-KEY(“Therapy Exercise”) OR TITLE-ABS-KEY(“Remedial Exercise”) OR TITLE-ABS-KEY(“Remedial Exercises”) OR TITLE-ABS-KEY(“exercise therapy”) OR TITLE-ABS-KEY(“Training Exercise”) OR TITLE-ABS-KEY(“Exercise Training”) OR TITLE-ABS-KEY(“Exercise Trainings”) OR TITLE-ABS-KEY(“Aerobic Exercise”) OR TITLE-ABS-KEY(“Aerobic Exercises”) OR TITLE-ABS-KEY(“Exercise Aerobic”) OR TITLE-ABS-KEY(“Exercises Aerobic”) OR TITLE-ABS-KEY(“Isometric Exercise”) OR TITLE-ABS-KEY(“Isometric Exercises”) OR TITLE-ABS-KEY(“Exercise Isometric”) OR TITLE-ABS-KEY(“Exercises Isometric”) OR TITLE-ABS-KEY(“Exercise Acute”) OR TITLE-ABS-KEY(“Acute Exercise”) OR TITLE-ABS-KEY(“Acute Exercises”) OR TITLE-ABS-KEY(“Physical Exercise”) OR TITLE-ABS-KEY(“Physical Exercises”) OR TITLE-ABS-KEY(“Exercise Physical”) OR TITLE-ABS-KEY(“Exercises Physical”) OR TITLE-ABS-KEY(“Physical Activities”) OR TITLE-ABS-KEY(“Activity Physical”) OR TITLE-ABS-KEY(“Physical Activity”) OR TITLE-ABS-KEY(Exercises) OR TITLE-ABS-KEY(exercise) OR TITLE-ABS-KEY(“Management Disease”) OR TITLE-ABS-KEY(“Disease Managements”) OR TITLE-ABS-KEY(“disease management”) OR TITLE-ABS-KEY(“drug therapy”) OR TITLE-ABS-KEY(“therapy drug”) OR TITLE-ABS-KEY(“drug therapies”) OR TITLE-ABS-KEY(Pharmacotherapy) OR TITLE-ABS-KEY(Pharmacotherapies) OR TITLE-ABS-KEY(intervention) OR TITLE-ABS-KEY(trial) OR TITLE-ABS-KEY(”mindfulness”) OR TITLE-ABS-KEY(”cognitive behavioral therapy”) OR TITLE-ABS-KEY(”cognitive-behavioral therapy”) OR TITLE-ABS-KEY(”self-management”) OR TITLE-ABS-KEY(”psychotherapy”) OR TITLE-ABS-KEY(”psychological treatment”) OR TITLE-ABS-KEY(”medication”)

**#2 Patients**

TITLE-ABS-KEY(“Libman Sacks Disease”) OR TITLE-ABS-KEY(“Libman-Sacks Disease”) OR TITLE-ABS-KEY(“Lupus Erythematosus Disseminatus”) OR TITLE-ABS-KEY(“Systemic Lupus Erythematosus”) OR TITLE-ABS-KEY(“Lupus Erythematosus Systemic”) OR TITLE-ABS-KEY(“Juvenile Myositis”) OR TITLE-ABS-KEY(Dermatopolymyositis) OR TITLE-ABS-KEY(“Dermatomyositis Juvenile”) OR TITLE-ABS-KEY(“Juvenile Dermatomyositis”) OR TITLE-ABS-KEY(“Childhood Type Dermatomyositis”) OR TITLE-ABS-KEY(“Polymyositis Dermatomyositis”) OR TITLE-ABS-KEY(Dermatomyositis) OR TITLE-ABS-KEY(Polymyositis-Dermatomyositis) OR TITLE-ABS-KEY(“Pediatric Rheumatic Conditions”) OR TITLE-ABS-KEY(“Juvenile Psoriatic Arthritis”) OR TITLE-ABS-KEY(“Juvenile Enthesitis-Related Arthritis”) OR TITLE-ABS-KEY(“arthritis juvenile”) OR TITLE-ABS-KEY(“juvenile arthritis”) OR TITLE-ABS-KEY(“Arthritis Juvenile Chronic”) OR TITLE-ABS-KEY(“Chronic Arthritis Juvenile”) OR TITLE-ABS-KEY(“Juvenile Rheumatoid Arthritis”) OR TITLE-ABS-KEY(“Juvenile Chronic Arthritis”) OR TITLE-ABS-KEY(“Arthritis Juvenile Rheumatoid”) OR TITLE-ABS-KEY(“Rheumatoid Arthritis Juvenile”) OR TITLE-ABS-KEY(“Juvenile Idiopathic Arthritis”) OR TITLE-ABS-KEY(“Still Disease”) OR TITLE-ABS-KEY(“Systemic Arthritis”) OR TITLE-ABS-KEY(“Arthritis Systemic”) OR TITLE-ABS-KEY(“Juvenile Systemic Arthritis”) OR TITLE-ABS-KEY(“Stills Disease”)

**#3 Outcome**

TITLE-ABS(Tiredness) OR TITLE-ABS(lassitude) OR TITLE-ABS(fatigue)

#1 AND #2 AND #3 AND limit to article AND child OR adolescents

**Embase**

**#1 intervention**

“Drug therapy”/exp OR "Therapeutics"/exp OR "Rehabilitation"/exp OR "Exercise"/exp OR "Disease Management"/exp OR "Exercise Therapy"/exp OR “strategies”:ti,ab,kw OR “response”:ti,ab,kw OR “remedy”:ti,ab,kw OR “regime”:ti,ab,kw OR “recovery”:ti,ab,kw OR “program”:ti,ab,kw OR “programs”:ti,ab,kw OR “management”:ti,ab,kw OR “manage”:ti,ab,kw OR “intervention”:ti,ab,kw OR “help”:ti,ab,kw OR “healing”:ti,ab,kw OR “doctoring”:ti,ab,kw OR “curing”:ti,ab,kw OR “cure”:ti,ab,kw OR “care”:ti,ab,kw OR “aid”:ti,ab,kw OR “training”:ti,ab,kw OR “trainings”:ti,ab,kw OR “Treatments”:ti,ab,kw OR “Treatment”:ti,ab,kw OR “Therapies”:ti,ab,kw OR “Therapy”:ti,ab,kw OR “therapeutic”:ti,ab,kw OR “therapeutics”:ti,ab,kw OR “habilitation”:ti,ab,kw OR “rehabilitation”:ti,ab,kw OR “Procedure”:ti,ab,kw OR “procedures”:ti,ab,kw OR “Exercise Rehabilitation”:ti,ab,kw OR “Exercises Rehabilitation”:ti,ab,kw OR “Rehabilitation Exercise”:ti,ab,kw OR “Rehabilitation Exercises”:ti,ab,kw OR “Therapies Exercise”:ti,ab,kw OR “Exercise Therapies”:ti,ab,kw OR “Therapy Exercise”:ti,ab,kw OR “Remedial Exercise”:ti,ab,kw OR “Remedial Exercises”:ti,ab,kw OR “exercise therapy”:ti,ab,kw OR “Training Exercise”:ti,ab,kw OR “Exercise Training”:ti,ab,kw OR “Exercise Trainings”:ti,ab,kw OR “Aerobic Exercise”:ti,ab,kw OR “Aerobic Exercises”:ti,ab,kw OR “Exercise Aerobic”:ti,ab,kw OR “Exercises Aerobic”:ti,ab,kw OR “Isometric Exercise”:ti,ab,kw OR “Isometric Exercises”:ti,ab,kw OR “Exercise Isometric”:ti,ab,kw OR “Exercises Isometric”:ti,ab,kw OR “Exercise Acute”:ti,ab,kw OR “Acute Exercise”:ti,ab,kw OR “Acute Exercises”:ti,ab,kw OR “Physical Exercise”:ti,ab,kw OR “Physical Exercises”:ti,ab,kw OR “Exercise Physical”:ti,ab,kw OR “Exercises Physical”:ti,ab,kw OR “Physical Activities”:ti,ab,kw OR “Activity Physical”:ti,ab,kw OR “Physical Activity”:ti,ab,kw OR “Exercises”:ti,ab,kw OR “exercise”:ti,ab,kw OR “Management Disease”:ti,ab,kw OR “Disease Managements”:ti,ab,kw OR “disease management”:ti,ab,kw OR “drug therapy”:ti,ab,kw OR “therapy drug”:ti,ab,kw OR “drug therapies”:ti,ab,kw OR “Pharmacotherapy”:ti,ab,kw OR “Pharmacotherapies”:ti,ab,kw OR “intervention”:ti,ab,kw OR “trial”:ti,ab,kw OR **"mindfulness"**:ti,ab,kw OR **"cognitive behavioral therapy"**:ti,ab,kw **OR "cognitive-behavioral therapy"**:ti,ab,kw **OR "self-management"**:ti,ab,kw **OR "psychotherapy"**:ti,ab,kw **OR "psychological treatment"**:ti,ab,kw **OR "medication"**:ti,ab,kw

**#2 Patients**

"Lupus Erythematosus, Systemic"/exp OR "Dermatomyositis"/exp OR "Arthritis, Juvenile"/exp OR “Libman Sacks Disease”:ti,ab,kw OR “Libman-Sacks Disease”:ti,ab,kw OR “Lupus Erythematosus Disseminatus”:ti,ab,kw OR “Systemic Lupus Erythematosus”:ti,ab,kw OR “Lupus Erythematosus Systemic”:ti,ab,kw OR “Juvenile Myositis”:ti,ab,kw OR “Dermatopolymyositis”:ti,ab,kw OR “Dermatomyositis Juvenile”:ti,ab,kw OR “Juvenile Dermatomyositis”:ti,ab,kw OR “Childhood Type Dermatomyositis”:ti,ab,kw OR “Polymyositis Dermatomyositis”:ti,ab,kw OR “Dermatomyositis”:ti,ab,kw OR “Polymyositis-Dermatomyositis”:ti,ab,kw OR “Pediatric Rheumatic Conditions”:ti,ab,kw OR “Juvenile Psoriatic Arthritis”:ti,ab,kw OR “Juvenile Enthesitis-Related Arthritis”:ti,ab,kw OR “arthritis juvenile”:ti,ab,kw OR “juvenile arthritis”:ti,ab,kw OR “Arthritis Juvenile Chronic”:ti,ab,kw OR “Chronic Arthritis Juvenile”:ti,ab,kw OR “Juvenile Rheumatoid Arthritis”:ti,ab,kw OR “Juvenile Chronic Arthritis”:ti,ab,kw OR “Arthritis Juvenile Rheumatoid”:ti,ab,kw OR “Rheumatoid Arthritis Juvenile”:ti,ab,kw OR “Juvenile Idiopathic Arthritis”:ti,ab,kw OR “Still Disease”:ti,ab,kw OR “Systemic Arthritis”:ti,ab,kw OR “Arthritis Systemic”:ti,ab,kw OR “Juvenile Systemic Arthritis”:ti,ab,kw OR “Stills Disease”:ti,ab,kw

**#3 Outcome**

“Tiredness”:ti,ab,kw OR “lassitude”:ti,ab,kw OR “fatigue”:ti,ab,kw

#1 AND #2 AND #3 AND Infant OR Child OR preschool child OR school child OR adolescent AND article AND Controlled study.

**Pedro**

Lupus AND Paediatrics.

Myositis AND Paediatrics.

Dermatopolymyositis AND Paediatrics.

Dermatomyositis AND paediatrics.

Arthritis AND paediatrics.

**Appendix II**

Revised Cochrane risk-of-bias tool for randomized trials (RoB 2.0)

TEMPLATE FOR COMPLETION

Edited by Julian PT Higgins, Jelena Savović, Matthew J Page, Jonathan AC Sterne
on behalf of the ROB2 Development Group

**Version of 9 October 2018**

The development of the RoB 2 tool was supported by the MRC Network of Hubs for Trials Methodology Research (MR/L004933/2- N61), with the support of the host MRC ConDuCT-II Hub (Collaboration and innovation for Difficult and Complex randomised controlled Trials In Invasive procedures - MR/K025643/1), by MRC research grant MR/M025209/1, and by a grant from The Cochrane Collaboration.


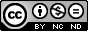


This work is licensed under a [Creative Commons Attribution-NonCommercial-NoDerivatives 4.0 International License](http://creativecommons.org/licenses/by-nc-nd/4.0/).

| **Study details**   \| **Reference** \|  \| \| --- \| --- \|   **Study design**   \| □ \| Individually-randomized parallel-group trial \| \| --- \| --- \| \| □ \| Cluster-randomized parallel-group trial \| \| □ \| Individually randomized cross-over (or other matched) trial \|  \| **Specify which outcome is being assessed for risk of bias** \|  \| \| --- \| --- \|  \| **Specify the numerical result being assessed.** In case of multiple alternative analyses being presented, specify the numeric result (e.g. RR = 1.52 (95% CI 0.83 to 2.77) and/or a reference (e.g. to a table, figure or paragraph) that uniquely defines the result being assessed. \|  \| \| --- \| --- \|   **Is the review team’s aim for this result…?**   \| □ \| to assess the effect of *assignment to intervention* (the ‘intention-to-treat’ effect) \| \| --- \| --- \| \| □ \| to assess the effect of *adhering to intervention* (the ‘per-protocol’ effect) \|   **Which of the following sources were obtained to help inform the risk-of-bias assessment? (tick as many as apply)**  □ Journal article(s) with results of the trial  □ Trial protocol  □ Statistical analysis plan (SAP)  □ Non-commercial trial registry record (e.g. ClinicalTrials.gov record)  □ Company-owned trial registry record (e.g. GSK Clinical Study Register record)  □ “Grey literature” (e.g. unpublished thesis)  □ Conference abstract(s) about the trial  □ Regulatory document (e.g. Clinical Study Report, Drug Approval Package)  □ Research ethics application  □ Grant database summary (e.g. NIH RePORTER or Research Councils UK Gateway to Research)  □ Personal communication with trialist  □ Personal communication with the sponsor |
| --- | --- | --- | --- | --- | --- | --- | --- | --- | --- | --- | --- | --- | --- | --- | --- | --- |

**Domain 1: Risk of bias arising from the randomization process**

| **Signalling questions** | **Description** | **Response options** |
| --- | --- | --- |
| **1.1 Was the allocation sequence random?** |  | Y / PY / PN / N / NI |
| **1.2 Was the allocation sequence concealed until participants were enrolled and assigned to interventions?** |  | Y / PY / PN / N / NI |
| **1.3 Did baseline differences between intervention groups suggest a problem with the randomization process?** |  | Y / PY / PN / N / NI |
| **Risk-of-bias judgement** |  | Low / High / Some concerns |
| Optional: What is the predicted direction of bias arising from the randomization process? |  | Favours experimental / Favours comparator / Towards null /Away from null / Unpredictable |

**Domain 2: Risk of bias due to deviations from the intended interventions (*effect of assignment to intervention*)**

| **Signalling questions** | **Description** | **Response options** |
| --- | --- | --- |
| **2.1. Were participants aware of their assigned intervention during the trial?** |  | Y / PY / PN / N / NI |
| **2.2. Were carers and people delivering the interventions aware of participants' assigned intervention during the trial?** |  | Y / PY / PN / N / NI |
| **2.3. If Y/PY/NI to 2.1 or 2.2: Were there deviations from the intended intervention that arose because of the experimental context?** |  | NA / Y / PY / PN / N / NI |
| **2.4. If Y/PY to 2.3: Were these deviations from intended intervention balanced between groups?** |  | NA / Y / PY / PN / N / NI |
| **2.5 If N/PN/NI to 2.4: Were these deviations likely to have affected the outcome?** |  | NA / Y / PY / PN / N / NI |
| **2.6 Was an appropriate analysis used to estimate the effect of assignment to intervention?** |  | Y / PY / PN / N / NI |
| **2.7 If N/PN/NI to 2.6: Was there potential for a substantial impact (on the result) of the failure to analyse participants in the group to which they were randomized?** |  | NA / Y / PY / PN / N / NI |
| **Risk-of-bias judgement** |  | Low / High / Some concerns |
| Optional: What is the predicted direction of bias due to deviations from intended interventions? |  | Favours experimental / Favours comparator / Towards null /Away from null / Unpredictable |

**Domain 3: Missing outcome data**

| **Signalling questions** | **Description** | **Response options** |
| --- | --- | --- |
| **3.1 Were data for this outcome available for all, or nearly all, participants randomized?** |  | Y / PY / PN / N / NI |
| **3.2 If N/PN/NI to 3.1: Is there evidence that result was not biased by missing outcome data?** |  | NA / Y / PY / PN / N |
| **3.3 If N/PN to 3.2: Could missingness in the outcome depend on its true value?** |  | NA / Y / PY / PN / N / NI |
| **3.4 If Y/PY/NI to 3.3: Do the proportions of missing outcome data differ between intervention groups?** |  | NA / Y / PY / PN / N / NI |
| **3.5 If Y/PY/NI to 3.3: Is it likely that missingness in the outcome depended on its true value?** |  | NA / Y / PY / PN / N / NI |
| **Risk-of-bias judgement** |  | Low / High / Some concerns |
| Optional: What is the predicted direction of bias due to missing outcome data? |  | Favours experimental / Favours comparator / Towards null /Away from null / Unpredictable |

**Domain 4: Risk of bias in measurement of the outcome**

| **Signalling questions** | **Description** | **Response options** |
| --- | --- | --- |
| **4.1 Was the method of measuring the outcome inappropriate?** |  | Y / PY / PN / N / NI |
| **4.2 Could measurement or ascertainment of the outcome have differed between intervention groups ?** |  | Y / PY / PN / N / NI |
| **4.3 If N/PN/NI to 4.1 and 4.2: Were outcome assessors aware of the intervention received by study participants ?** |  | Y / PY / PN / N / NI |
| **4.4 If Y/PY/NI to 4.3: Could assessment of the outcome have been influenced by knowledge of intervention received?** |  | NA / Y / PY / PN / N / NI |
| **4.5 If Y/PY/NI to 4.4:** **Is it likely that assessment of the outcome was influenced by knowledge of intervention received?** |  | NA / Y / PY / PN / N / NI |
| **Risk-of-bias judgement** |  | Low / High / Some concerns |
| Optional: What is the predicted direction of bias in measurement of the outcome? |  | Favours experimental / Favours comparator / Towards null /Away from null / Unpredictable |

**Domain 5: Risk of bias in selection of the reported result**

| **Signalling questions** | **Description** | **Response options** |
| --- | --- | --- |
| **5.1 Was the trial analysed in accordance with a pre-specified plan that was finalized before unblinded outcome data were available for analysis ?** |  | Y / PY / PN / N / NI |
| **Is the numerical result being assessed likely to have been selected, on the basis of the results, from...** |  |  |
| **5.2. ... multiple outcome measurements (e.g. scales, definitions, time points) within the outcome domain?** |  | Y / PY / PN / N / NI |
| **5.3 ... multiple analyses of the data?** |  | Y / PY / PN / N / NI |
| **Risk-of-bias judgement** |  | Low / High / Some concerns |
| Optional: What is the predicted direction of bias due to selection of the reported result? |  | Favours experimental / Favours comparator / Towards null /Away from null / Unpredictable |

**Overall risk of bias**

| **Risk-of-bias judgement** |  | Low / High / Some concerns |
| --- | --- | --- |
| Optional: What is the predicted direction of bias due to selection of the reported result? |  | Favours experimental / Favours comparator / Towards null /Away from null / Unpredictable |

**Appendix III.** *The quality assessment tool for before-after (pre-post) studies with no control group.*

|  |  | Scale Items^a^ | | | | | | | | | | | | |
| --- | --- | --- | --- | --- | --- | --- | --- | --- | --- | --- | --- | --- | --- | --- |
|  | | 1 | 2 | 3 | 4 | 5 | 6 | 7 | 8 | 9 | 10 | 11 | 12 | Score |
| Author, year | |  |  |  |  |  |  |  |  |  |  |  |  |  |

^a^ Refer to table below for criteria

The quality assessment tool for before-after (pre-post) studies with no control group: criteria.

| Criteria | Scale Items |
| --- | --- |
| Was the study question or objective clearly stated? | 1 |
| Were eligibility/selection criteria for the study population pre-specified and clearly described? | 2 |
| Were the participants in the study representative of those who would be eligible for the test/service/intervention in the general or clinical population of interest? | 3 |
| Were all eligible participants that met the pre-specified entry criteria enrolled? | 4 |
| Was the sample size sufficiently large to provide confidence in the findings? | 5 |
| Was the test/service/intervention clearly described and delivered consistently across the study population? | 6 |
| Were the outcome measures pre-specified, clearly defined, valid, reliable, and assessed consistently across all study participants? | 7 |
| Were the people assessing the outcomes blinded to the participants' exposures/interventions? | 8 |
| Was the loss to follow-up after baseline 20% or less? Were those lost to follow-up accounted for in the analysis? | 9 |
| Did the statistical methods examine changes in outcome measures from before to after the intervention? Were statistical tests done that provided p values for the pre-to-post changes? | 10 |
| Were outcome measures of interest taken multiple times before the intervention and multiple times after the intervention (i.e., did they use an interrupted time-series design)? | 11 |
| If the intervention was conducted at a group level (e.g., a whole hospital, a community, etc.) did the statistical analysis take into account the use of individual-level data to determine effects at the group level?  *If this question is not applicable, total score is out of 11, not 12. | 12 |
| Add scores for each criterion together and divide by 12.  *Risk of bias rating (Low (75-100%), Moderate (25-75%), or High (0-25%))**  OVERALL SCORE: |  |

# *This section includes altered wording from original tool for consistency purposes

Key: Y = Yes, N = No, NR = Not reported, CD = Cannot determine, NA = Not applicable, M = Moderate

**Appendix IV.** Risk of bias summary: review authors’ judgements about each risk of bias item for each included controlled study.

| *Author, year (ref.)*  *Study design* | 1. Bias arising from the randomization process | 2. Bias due to deviations from intended interventions | 3. Bias due to missing outcome data | 4. Bias in measurement of the outcome | 5. Bias in selection of the reported result | Overall risk of bias |
| --- | --- | --- | --- | --- | --- | --- |
| *Habers,*  *2016* (27)  *RCT* |  |  |  |  |  |  |
| *Sule, 2019 (sule)*  *RCT* |  |  |  |  |  |  |
| *Lima,*  *2016* (30)  *RCT* |  |  |  |  |  |  |
| *Samhan, 2020* (35)  *CT* |  |  |  |  |  |  |
| *Kvien, 1982* (29)  *CT* |  |  |  |  |  |  |
| *Dover, 2020* (36)  *RCT* |  |  |  |  |  |  |
| *Fuchs, 2013* (31)  *RCT* |  |  |  |  |  |  |
| *Hilderson,*  *2016* (32)  *CT* |  |  |  |  |  |  |
| CT: controlled trial, RCT: randomized controlled trial, ref.: reference  : high risk of bias,: some concerns/ moderate risk of bias,  : low risk of bias. | | | | | | |

**Appendix V.** Risk of bias summary: the quality assessment scores of the included pre-post studies.

|  | Question clear? | Selection criteria clear? | Participants representative? | All eligible participants enrolled? | Sample size sufficiently large? | Intervention clearly described? | Outcome measure accurate? | Assessors blinded? | Lost to follow-up 20% or less? | Statistical methods accurate? | Interrupted time- series design? | Individual-level data used? | Total score |
| --- | --- | --- | --- | --- | --- | --- | --- | --- | --- | --- | --- | --- | --- |
| *Houghton 2018* (28) | Y | Y | Y | N | N | Y | Y | N | N | Y | N | Y | 7/12=58% |
| *Cunningham 2019(ref)* | Y | Y | Y | N | N | N | Y | NR | N | Y | N | N | 5/12=42% |
| N: no, Y: yes, : some concerns/moderate risk of bias. | | | | | | | | | | | | | |

**Appendix VI: Results of individual studies**

*Effectiveness of exercise therapy*

One multi-centre RCT (27), with moderate risk of bias, has examined the effects of exer­cise training in JDM. Twenty-six patients (age 8.3 to 17.6 years) were allo­cated to either an exercise or control group; following a twelve-week individually tailored home-based exercise program of interval training on a treadmill and strength exercises. Of the participants, 75% completed the intervention. Patients who exercised showed greater improvements than controls in aerobic fitness, muscle function and functional ability, whereas perception of fatigue was unaffected (Δ (95% CI): 1 (-5.7), p=0.8).

One single-centre RCT (33), with high risk of bias, tested the effects of resistance exercise in a cohort of children with polyarticular JIA. Patients were allocated to either an exercise group (N=17, age 14±3.3 years) or a control group (N=16, age 16.1±2.8 years). The exercise group received slow speed resistance exercises with individualized instructions, 1-2 times per week for 12 weeks. The control group performed home bases aerobic exercises 3 days per week for 12 weeks. In the exercise group, 9/17 (53%) completed any exercise training. Of these nine subjects, five (55%) completed all 12 weeks of the protocol. In the control group, 8/16 (50%) reported compliance with the recommended aerobic exercise training at least one time per week. Only 2 subjects (12%) reported exercising more than once per week. There was no significant difference between pre- and post-measurement fatigue in the exercise or control group. There was also no significant difference in post measurements of fatigue severity scores between the exercise and control groups.

One pre-post exercise intervention trial (28), with moderate risk of bias, has examined the effects of a six-month home-based exercise program involving jumping and handgrip exercises, resistance training and one group session per month in children with JIA. Twenty-four participants (mean age of 13 years) volunteered to participate, whereof thirteen participants completed baseline, six- and twelve-month measures. Fatigue scores improved by 8.1 points and 12.0 points, on average, at 3 and 6 months, respectively, with some improvement maintained at 12 months (9.4 points) (p=0.034).

An assessor-blinded, controlled 2x2 crossover trial compared the effect of aquatic-based exercises (AQBEs) and land-based exercises (LBEs) on muscle strength, fatigue and QoL, and skin disease activity in children with JDM (35). Fourteen children (age 10 to 16 years) were assigned to one of two groups based on two treatment sequences children followed. The first group (n=7) received first treatment sequence starting by LBEs followed by AQBEs with a one-month washout period in-between, and the second group (n=7) received second treatment sequence which began with AQBEs followed by LBEs interspersed with a one-month washout period. There was a significant decrease in the general fatigue measured by PedsQL-MFS in both AQBEs and LBEs groups (F 3,4=517.6, p<0.001; Wilks’ Λ=0.003, partial η²=0.997). Nevertheless, AQBEs improved the PedsQL-MFS more than LBEs (baseline about 35, AQBEs improved to 61.9±3.9, LBEs improved to 44.2±2.3 (p<0.001)).

*Effectiveness of medication/nutritional supplements*

In one study, with high risk of bias (29), a parallel double-blind study of prednisolone vs placebo was carried out in twenty children with JRA (age 5 to 15.8 years). The intervention group received 0,4 mg/kg/day prednisolone for seven days and the control group received placebo tablets with identical appearance, smell and taste. Thirteen children completed the subjective assessment of fatigue. A significant improvement (p=0.03) was found for fatigue in the prednisolone group (median difference of 0, range -4,4) vs the placebo group (median difference of -1, range -6,0).

The effect of vitamin D supplementation was studied in one RCT (30). Forty adolescents were randomly assigned to the cSLE-Vitamin D group (age 18.5±3.5 years) or the cSLE-Placebo group (age 19.3±3.3 years). This study demonstrated that patients who received vitamin D supplementation (cholecalciferol 50,000 IU/week for six months) had a better global score for fatigue at the end of the study than the placebo group (3.15±1.44 versus 4.30±1.33, respectively; p=0.012).

The effect of creatine supplementation was studied in a 6-month double-blind, randomized, multiple-baseline design (36). Thirteen patients with JDM (age 7 to 14 years) randomly started creatine at one of 6 potential start times and was on creatine for a minimum of 4 weeks (maximum of 6 months), in order to achieve steady-state conditions. The subjects were prescribed creatine/placebo based on their weight, up to 40kg, or their body surface area for those weighing >40kg at a dose of 150mg/kg/day or 4.69g/m2/day, in chewable, lemon-flavored tablets for 6 months. There were no statistically significant changes in fatigue between while subjects were on creatine compared to on placebo (mean difference 3.6, p=0.982).

*Effectiveness of psychological intervention/a transition program*

The effect of a psychological intervention with the Self-confrontation Method in adolescents with JIA (age 16.1±1.7 years) was studied in one partly RCT with high risk of bias (31). In phase two of the intervention eighteen adolescents with JIA were randomly assigned to the six- or twelve-session group. The twelve-session group received six-weekly individual sessions of about one hour each extra. After analysis of the results at three months of the two different groups, no significant differences were observed in fatigue. Therefore, because the total sample size was modest, the separation was left out of further analysis. They state that the intensity of the intervention have no influence on the outcome measures. In general, adolescents with JIA already benefit from modest psychological intervention.

One study aimed to develop and refine the Treatment and Education Approach for Childhood-onset Lupus (TEACH) protocol, a brief cognitive behavioral intervention specifically tailored for adolescents and young adults with cSLE, and assessed preliminary evidence of feasibility, acceptability, and impact on symptoms of fatigue, psychological distress and pain (34). Fourteen participants (age 16.21±2.05 years) completed six weekly in-person sessions facilitated by a doctoral level psychologist. During the study, several protocol modifications were employed to better address the unique needs of individuals with cSLE (e.g., separate content for adolescents versus young adults). Results suggest that TEACH is feasible, acceptable, and potentially effective in the management of cSLE symptoms. Following the intervention, there was a statistically significant reduction in fatigue (Average decrease of 5.40, Z=−2.81, p=0.005).

The clinical impact of a brief transition program for young people with JIA was investigated in a quasi-experimental study with a post-test-only comparison group design, with high risk of bias (32). The transition program comprised eight key components, among which a transition coordinator, education, information, guidance of parents, meeting with peers, a transfer plan and the actual transfer to an adult rheumatology program. Twenty-three participants from the intervention group (age 16.6 to 18.7 years) could be matched with a participant in the comparison group (age 17.5 to 20.2 years). The participants of the comparison group had already been transferred to the adult rheumatology program without participating in a specific self-management/transition program. A small positive effect was found in reduction in mental fatigue (ES=0.28), general fatigue (ES=0.27) and physical fatigue (ES=0.22) and in increasing activity (ES=0.27) and motivation (ES=0.23).

**Appendix VII.** Conceptual model of fatigue in patients with JIA (6).


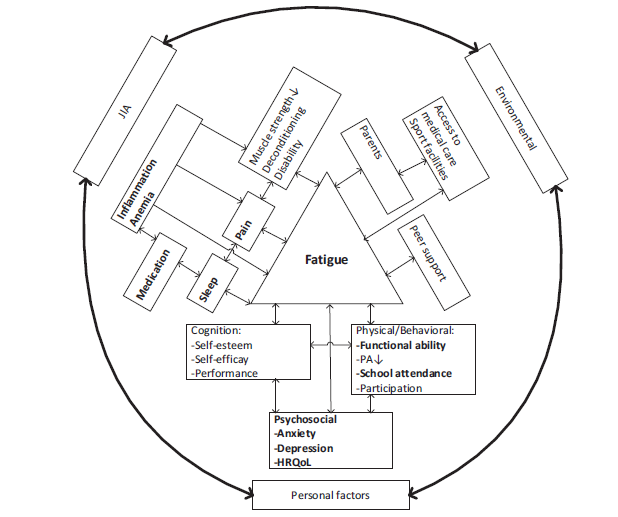


*↓unidirectional relation, ↕ bidirectional relation, items in bold are known from the literature to be correlated with fatigue in patients with JIA.*
